# Analysis of 6 pediatric nephrotic syndrome cases with complications of cerebral sinovenous thrombosis and literature review

**DOI:** 10.3389/fped.2023.1226557

**Published:** 2023-09-11

**Authors:** Xuan Lu, Cao Yan, Hui Chen, Xiaochuan Wu

**Affiliations:** Second Xiangya Hospital, Central South University, Changsha, China

**Keywords:** sinovenous thrombosis, nephrotic syndrome, children, transient receptor potential channel 6, clinical characteristic

## Abstract

**Background:**

Cerebral venous sinus thrombosis (CVST) is a rare but serious complication of nephrotic syndrome (NS) in children. To investigate the clinical characteristics of CVST in children with NS in order to timely diagnose this complication and reduce poor outcome.

**Methods:**

Collect and analyze clinical data and magnetic resonance venography (MRV) results of children with NS complicated with CVST.

**Results:**

Data of 6 patients with NS complicated with CVST were collected. 4 of the patients were steroid-sensitive nephrotic syndrome (SSNS) and 2 were steroid-resistant nephrotic syndrome (SRNS). The occurrence of CVST was observed within a time frame ranging from 12 days to 3 years following the diagnosis of NS. One patient had two episodes of thrombosis in three years, while the other five patients had only one episode of thrombosis. All patients had proteinuria at the time of episode of thrombosis. All patients presented with headache, and three of them had strabismus, seizures, and transient blindness, respectively. Neurological examination was negative. All patients were diagnosed with CVST by MRV within 3–16 days of the onset of headache. Two patients had TRPC6 gene mutation. All patients had resolution of neurological symptoms after anticoagulation treatment.

**Conclusion:**

CVST may occur in the early stages of NS. There is currently a lack of specific diagnostic indicators to reliably identify the presence of CVST in patients with NS. Children with NS who have neurological symptoms should be promptly evaluated with imaging studies. Whether TRPC6 gene mutation is also a risk factor for CVST remains to be further studied.

## Introduction

Thromboembolism is a rare but serious complication of nephrotic syndrome (NS) in children. A study by the Midwest Pediatric Nephrotic Society (MWPNC) showed that the incidence of thromboembolism in children with primary nephrotic syndrome (PNS) was 6.6% and the incidence in secondary NS is 17.1%. Deep vein thrombosis is the most common type of thromboembolism in children with nephrotic syndrome, accounting for 76% of cases (1). Compared to other types of thromboembolism, CVT has a lower incidence, accounting for only 2%–5% of cases (2). The symptoms and signs of CVST are variable and nonspecific, patients may present as headache, vomit due to raised intracranial pressure or signs with focal neurological deficits. The diagnosis of thrombotic events based on laboratory indicators is controversial, so the diagnosis of CVST currently relies on imaging examinations. However, not all patients can receive timely imaging examinations, which can delay the diagnosis of CVST. Venous blockage that is not treated promptly can progress to venous infarction or cerebral hemorrhage, which can have serious consequences. However, patients who receive timely anticoagulant therapy typically have a good prognosis and low mortality (3, 4).

## Methods

### Patients

The study subjects were children with nephrotic syndrome (NS) complicated by cerebral venous sinus thrombosis (CVST) who were admitted to the Department of Pediatrics of the Second Xiangya Hospital Affiliated to Central South University from January 2014 to December 2022. The diagnosis of NS was based on the following criteria: (1) massive albuminuria: 24-hour urinary protein quantity ≥50 mg/kg; (2) hypoalbuminemia: serum albumin <25 g/L; (3) hyperlipidemia; and (4) edema. All patients received magnetic resonance venography (MRV) to confirm CVST.

### Clinical data

The collected data included general characteristics and clinical data, such as the course of NS, response to steroids, number of thrombotic events, clinical manifestations, and thrombotic site. Laboratory results included urinary protein, serum albumin, cholesterol, and D-dimer values. Imaging data included CT, MR, and MRI. Therapy, efficacy, and follow-up were also recorded.

## Result

### General data

Five of the six children were female, aged from 6 to 16 years, and the duration of their disease ranged from 12 days to 3 years. One child had a second seizure. Two of them had steroid-resistant nephrotic syndrome (SRNS). All cases occurred during disease recurrence. Four patients had multiple venous sinuses involved, and two patients had venous infarction. Detailed information is shown in Table 1.

**Table 1:** General data of six cases.

Case	Course	Diagnosis	Clinical manifestation	Time to delay diagnosis
1	12 days	SRNS	Headache, diplopia, posterior esotropia	16 days
2	6 months	SSNS(relapse)	Headache	4 days
3	46 days	SSNS	Headache, epilepsy	3 days
4	11 days	SSNS	Hemiplegia	3 days
	3 years	SSNS(relapse)	Headache, transient blindness	4 days
5	1 years	SSNS	Headache, vomit	12 days
6	6 months	SRNS	Headache, vomit	13 days

Course: The duration between the diagnosis of nephrotic syndrome and the onset of symptoms related to cerebral venous sinus thrombosis.

Time to delay diagnosis: The interval between the onset of CVST symptoms and the diagnosis of CVST.

### Manifestations

All patients presented with headache, and three of them had strabismus, seizures, hemiplegia and transient blindness, respectively. Neurological examination was negative.

### Laboratory tests

All six cases were in the recurrent stage of nephrotic syndrome (NS) when they developed cerebral venous sinus thrombosis (CVST). D-dimer was elevated in all patients, with an average of 5.97 micrograms per milliliter. Antithrombin III (AT-III) showed a significant decrease in only two cases (43% and 31%), while it was nearly normal in the others. (See Table 2 for more details. Data for case 4 are from her second thrombotic event, as the data from her first event in another hospital are unknown.) All patients were tested for protein C and protein S activity to rule out congenital thrombophilia. Lumbar puncture examination showed that all patients had increased intracranial pressure (up to 400 mmHg), and cerebrospinal fluid examination showed no obvious abnormalities. Two cases with steroid-resistant disease underwent genetic testing, and the results both showed a TRCP6 mutation (c. 523C > T).

**Table 2:** Lab tests of six cases.

Case	Urine protein (g/24 h)	Serum cholesterol (mmol/L)	Serum albumin (g/L)	D-dimer (mg/L)	AT-III (%)
1	3.0	13.61	20.2	2.56	116
2	7.4	10.2	13.7	7.0	68
3	10.5	11.9	13.8	1.1	96
4	11.1	7.3	18.1	2.0	43
5	5.5	13.2	10.4	20.6	31
6	6.4	7.6	19.2	2.6	103
Mean	7.30	10.63	15.90	5.97	76.10

### Imaging examination

**Figure 1:**
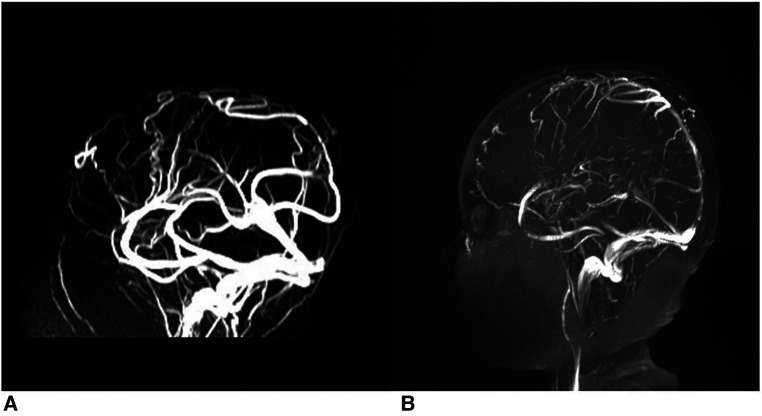
Thrombi in superior sagittal sinus of case 5. (**A, B**) Depict the comparison of pre- and post-administration of a two-week anticoagulation therapy.

Two patients received head CT examinations on admission, but no abnormalities were found. Four patients underwent head MRI examinations alone, three of which suggested the possibility of venous sinus embolism, and the other two reported no abnormalities and linear meningeal enhancement, respectively. All cases were diagnosed by MRV examination, and case 5 reported that the superior sagittal sinus was not visualized throughout (Figure 1). The shortest delay in diagnosis was 3 days. The longest case, case 1, was misdiagnosed as “viral encephalitis” in another hospital and was only diagnosed after being admitted to our hospital. Embolic infarctions were seen in the MRIs of two children, who developed diplopia and hemiplegia, respectively. None of the patients received digital subtraction angiography (DSA). The positive rates of CT, MRI, and MRA were 0% (0/2), 50% (2/4), and 100% (6/6), respectively.

### Treatment and outcome

After diagnosis, all cases were treated with heparin anticoagulation and other symptomatic treatment. They were then gradually transitioned to oral anticoagulant drug warfarin. The international normalized ratio (INR) was regularly monitored to adjust the drug dosage. After treatment, the symptoms were significantly improved in all children.

## Discussion

Thrombosis in the cerebral venous sinus (CSVT) is a relatively rare condition, with an estimated incidence of 1.8%–5.0% in China (5). Simultaneously, the manifestations of CVST exhibit variability and a dearth of specificity, particularly among pediatric patients who may struggle to articulate their sensations, thereby augmenting the diagnostic challenge. Consequently, certain scholars posit that the prevalence of CVST could potentially be underestimated (6). Focal neurological deficits, such as hemiplegia, hemianopsia, and seizures, can occur in CSVT. However, the most common presentation is simple isolated intracranial hypertension (ICH). Headache is a common symptom in children with CSVT, with 75% of patients reporting headache that is not relieved with rest or medication (7). The headache in children with CSVT can be variable in degree and nature. In this study, it was observed that a solitary child experienced persistent severe headache, prompting immediate imaging that revealed occlusion of the superior sagittal sinus. Conversely, the remaining children in this study only had headache that was irregular and intermittent headache. In both children and adults, simple intracranial hypertension type CSVT may be delayed in diagnosis due to lack of attention (8). In addition, ocular hypertension, hypertension, and concomitant infection, which are common in children with NS, can also interfere with the diagnosis. Three cases in this study had infection symptoms of sore throat, cough, and runny nose, and one child had slightly higher intraocular pressure (left eye 21 mmHg and right eye 21 mmHg). Both infection and high intraocular pressure could explain the headache, but they were completely different from CSVT in terms of imaging and treatment.

It is worth noting that CVST can occur in the early stage of the disease. Akatsu analyzed previous cases and found that the median course of the disease was 71 days before CVST occurred (9). Fluss believes that most CVST occurs within 6 months of the diagnosis of NS in children (10). The median course of NS in the cases presented in this literature was 46 days, indicating that CVST should be considered in the early stage of NS. To efficiently and cost-effectively differentiate patients with cerebral venous sinus thrombosis (CVST) from those presenting symptoms of intracerebral hemorrhage, physicians have undertaken a series of inquiries referring to indicators such as clot-related laboratory assessments and the presence of an underlying primary condition. Heldner et al. divided the patients with headache into low, medium, and high probability groups according to clinical manifestations, past medical history, and other data. In the low-risk group, the sensitivity and specificity of D-dimer levels exceeding 500 µg/L were both observed to be 100%. But the findings indicated that the prognostic efficacy of D-dimer in predicting cerebral venous thrombosis was comparatively diminished in the medium and high risk group than as opposed to the low-risk group (11). In other words, the predictive value of D-dimer elevation for intracranial thrombosis is limited among patients with NS in high-risk group. In general, diagnostic value of commonly used indicators for thrombosis in patients with nephrotic syndrome is limited due to the presence of low albumin, elevated D-dimer, and decreased AT-III levels resulting from pathological characteristics. Currently, there are no specific indicators available to accurately diagnose therombus occurrence of thrombus in patients with nephrotic syndrome (12–14).

Two patients underwent genetic testing because they had a poor response to immunosuppressive therapy. The results revealed *de novo* TRPC6 mutations. TRPC6 mutations, most of which are gain-of-function, are associated with familial late-onset FSGS and sporadic podocyte-associated nephropathy (15–17). Espinosa observed prolonged bleeding time and thrombosis time in TRPC6-deficient mice (18). Mahaut-Smith suggested that the co-activation of TRPC6 and TRPC3 increased cytosolic calcium, which triggered the exposure of phosphatidylserine (PS) on the platelet surface and accelerated the generation of thrombin (19). In this study, two cases of the same mutation [TRPC6 (p.R175W)] were detected in children with SRNS. In this study, two cases of the same mutation [TRPC6 (p.R175W)] were detected in children with SRNS. Hofstra et al. used patch clamp analysis to test the function of different missense mutations (p.R175Q) on the same amino acid residue, and the results showed that they were also gainful mutations (20). Since TRPC6 may have a role in promoting thrombosis, it is possible that children with NS who have this mutation are more likely to develop thrombosis than children with NS who do not have the mutation. However, a large sample size study is still lacking to confirm this hypothesis.

Due to the above reasons, the diagnosis of CVST in children mainly depends on imaging. However, there are still many misdiagnoses and missed diagnoses in clinical practice, even with the availability of more sophisticated imaging techniques. This is because the venous sinus is close to the dura mater, and it can sometimes be mistaken for subarachnoid hemorrhage or arachnoid granulation on imaging (21, 22). Plain CT is the first-line imaging modality for headache, but it has a high missed diagnosis rate of up to 30%–40% for CVST in children (23). In this paper, two of the four children who underwent head CT did not show any abnormalities. This suggests that relying solely on CT can lead to delayed diagnosis of CVST in children. So receiving only CT examination partially led to prolonged diagnosis delay in the child with CVST. MRI is superior to CT for demonstrating venous thrombosis. MRV can more visually show the filling of the cerebral venous sinus. It can show discontinuous, heterogeneous low blood flow signals or even no blood flow signals, depending on the degree of stenosis of the embolic venous sinus. This helps to compensate for the false-negative rate of MRI (21). In addition to focusing on the direct signs of a clot, assessing any damage to the cerebral parenchyma secondary to the thrombosis by looking for signs of hemorrhage or infarction can also be helpful for the diagnosis of CVST. All of the children in this study were ultimately diagnosed with CVST by MRI and MRV.

Currently, anticoagulant therapy is considered to be safe and effective for CVST. In this article, the children received heparin sodium for anticoagulation and urokinase for thrombolysis, and their symptoms improved quickly. At present, it is believed that anticoagulant therapy for CVST is safe and effective. In this article, the children received heparin for anticoagulation and the symptoms relieved quickly. For example, the initial severe headache of the child with massive thrombosis of the superior sagittal sinus was significantly relieved after 2 weeks of anticoagulant therapy. At the same time, follow-up MRV showed that the negative area of the superior sagittal sinus was reduced and clear compared to the previous image. During the follow-up, CVST symptoms were completely relieved in all 6 patients, including those with diagnostic delay of more than 10 days, all of whom presented with isolated intracranial hypertension. Diagnostic delay was defined as the interval between the onset of CVST symptoms and the diagnosis of CVST. The median diagnostic delay was 7 days. Ichord believes that the diagnosis and treatment of CVST in children should also follow the principle of “time is brain.” Delayed diagnosis may cause cerebral parenchymal ischemia due to intracranial hypertension, which can then evolve into venous cerebral infarction, which is also an important risk factor for the poor outcome of CVST (24, 25). In general, children with CVST who receive timely anticoagulant therapy have a good prognosis.

Through the review of 6 NS children with CVST, the following experience can be drawn: when NS children have steroid resistance, intracranial hypertension symptoms, especially headache, CVST should be considered. Head MRI or even MRV should be performed in time to shorten the diagnostic delay time and achieve a good prognosis. Genetic testing should be considered for children with poor response to immunosuppressive therapy and thrombotic events in the early stage of the disease. More in-depth investigation is needed to explore whether TRPC6 gene mutation can result in a propensity for thrombosis.

## Data Availability

The original contributions presented in the study are included in the article/Supplementary Material, further inquiries can be directed to the corresponding author.
